# Effect of Electroacupuncture on Gut Microbiota in Participants With Knee Osteoarthritis

**DOI:** 10.3389/fcimb.2021.597431

**Published:** 2021-10-04

**Authors:** Tian-Qi Wang, Ling-Ru Li, Chun-Xia Tan, Jing-Wen Yang, Guang-Xia Shi, Li-Qiong Wang, Hui Hu, Zhi-Shun Liu, Jun Wang, Tong Wang, Yong Yuan, Wen-Rui Jia, Hua Li, Xin-Wei Wang, Bin Wu, Jian-Feng Tu, Cun-Zhi Liu

**Affiliations:** ^1^ International Acupuncture and Moxibustion Innovation Institute, School of Acupuncture-Moxibustion and Tuina, Beijing University of Chinese Medicine, Beijing, China; ^2^ National Institute of Traditional Chinese Medicine Constitution and Preventive Treatment of Diseases, Beijing University of Chinese Medicine, Beijing, China; ^3^ Department of Acupuncture and Moxibustion, Dongfang Hospital, Beijing University of Chinese Medicine, Beijing, China; ^4^ Department of Acupuncture and Moxibustion, Guang’an Men Hospital, China Academy of Chinese Medical Sciences, Beijing, China; ^5^ Department of Acupuncture and Moxibustion, Dongzhimen Hospital, Beijing University of Chinese Medicine, Beijing, China; ^6^ Department of Orthopedics, Institute of Acupuncture and Moxibustion, China Academy of Chinese Medical Sciences, Beijing, China

**Keywords:** electroacupuncture, gut microbiota, knee osteoarthritis, sham acupuncture, effect

## Abstract

A close relationship between knee osteoarthritis (KOA) and gut microbiota has recently been described. Herein, we aim to investigate the effect of electroacupuncture (EA) on gut microbiota in participants with KOA. We conducted a study of 60 participants with KOA and 30 matched healthy controls (HCs). Sixty participants were allocated to either EA group (n=30) or sham acupuncture (SA) group (n=30). Five obligatory acupoints and three adjunct acupoints were punctured in the EA group. Eight non-acupoints that were separated from conventional acupoints or meridians were used for the SA group. Participants in both groups received 24 sessions within eight weeks. Fecal microbial analyses by 16S ribosomal RNA gene sequencing were carried out after collecting stools at *T*_0_ and *T*_8_ weeks (Four samples with changed defecation habits were excluded). The results showed that both Western Ontario and McMaster Universities Osteoarthritis Index (WOMAC) total score (*P=*0.043) and NRS score (*P=*0.002) decreased more in EA group than those in SA group. Moreover, EA could reverse more KOA-related bacteria including *Bacteroides*, *[Eubacterium]_hallii_group*, *Agathobacte*r *and Streptococcus*. The number of significantly different genera between KOA patients and HCs were less after EA treatment than that after SA treatment. This meant that EA modified the composition of the gut microbiome, making it closer to healthy people, while not significantly affecting the microbial diversity. Two genera including *Agathobacter* (*P*=0.0163), *Lachnoclostridium* (*P*=0.0144) were statistically increased than baseline in EA group (paired Wilcoxon rank sum test). After EA treatment, *Bacteroides* (*P=*0.0394) was more abundant and *Streptococcus* (*P=*0.0306) was significantly reduced in patients who demonstrated adequate response than in those with inadequate response (Wilcoxon rank-sum test). Spearman correlation test between gut microbe and KOA clinical outcomes indicated that *Bacteroides* and *Agathobacter* was negatively correlated with NRS score, WOMAC total score, and WOMAC pain, stiffness and pain scores (*P*<0.001 or 0.05 or 0.01), while *Streptococcus* was positively correlated with them (*P*<0.05 or 0.01). Our study suggests that EA contributes to the improvement of KOA and gut microbiota could be a potential therapeutic target.

## Introduction

Knee osteoarthritis (KOA) is one of the most common chronic conditions and forms of arthritis worldwide, which features as a protracted course of disease, especially among elderly patients (Lawrence et al., 2008; Arc OGEN Consortium and arc OGEN Collaborators, 2012). KOA is the leading cause of lower extremity disability among older adults (Hunt and Takacs, 2014). The prevalence of symptomatic KOA was higher in women (10.3%) compared with men (5.7%) (Tang et al., 2016). With increasing life expectancy, osteoarthritis is anticipated to become the fourth leading cause of disability by the year 2020 (Hochberg et al., 2016).

The most common symptom of KOA patients is chronic knee pain, which leads to a decrease in the amount of activity, and the body of KOA patients is in a low-grade inflammatory state. In healthy humans, these intestinal microbes interact to maintain the stability of the intestinal microecology. It would produce corresponding symptoms and even disease once the intestinal microstability was imbalanced (Zackular et al., 2013). Its imbalance is an important trigger for the increase of inflammation level in the body, and it is also involved in the occurrence of KOA. According to clinical studies, patients who have adopted green-lipped mussels and glucosamine have improved the symptoms of KOA, and the structure of the gut microbiome has changed (Coulson et al., 2013). The role of the bacterial axis in the pathogenesis of KOA suggested that metabolic inflammation may accelerate the pathological process of KOA (Li et al., 2016). At the same time, follow-up studies suggested that the abundance of *Streptococcus* species was associated with increased knee pain, for this association was driven by local inflammation in the knee joint. After testing 256 taxonomies individually, Cindy G. Boer found a microbiome-wide association with knee WOMAC pain and *Streptococcus* spp., where greater *Streptococcus* spp. relative abundance is associated with higher knee WOMAC pain. The results indicated the microbiome is a possible therapeutic target for osteoarthritis-related knee pain (Boer et al., 2019).

Acupuncture, used in China and other Asian countries for the past 3,000 years, has the potential to manage chronic pain with effectiveness, especially among KOA (Astin et al., 2000). It was found that acupuncture can improve the diversity of gut microbiome and the content of beneficial flora through different acupoints, so as to achieve the purpose of adjusting gut microbiome. It is also recognized that the gut microbiome can have a profound influence on systemic inflammation and chronic disease (Hand et al., 2016). At present, there are few reports on whether acupuncture can improve knee joint function and reduce inflammatory response by regulating the amount and structure of gut microbiome in patients. In-depth discussion in this field has certain practical significance to reveal the internal mechanism of acupuncture to improve function and relieve pain of KOA.

## Methods

### Study Cohort

Sixty KOA participants were included from a large randomized clinical trial (Tu et al., 2021). All participants were recruited from five hospitals (Beijing Hospital of Traditional Chinese Medicine Affiliated to Capital Medical University, Dongzhimen Hospital of Beijing University of Chinese Medicine, Dongfang Hospital of Beijing University of Chinese Medicine, Guang’an Men Hospital and Institute of Acupuncture and Moxibustion) in Beijing, China. KOA participants were allocated to the electroacupuncture (EA) group or sham acupuncture (SA) group with 30 participants in each group. Fecal samples were collected twice before and after treatment. The inclusion criteria were as follows: age from 45 to 75; Kellgren-Lawrence (Kellgren and Lawrence, 1957) grade II or III (mild or moderate) radio graphically-confirmed KOA on one or both knees; duration of more than six months and pain intensity≥4 on a 10 numerical rating scale (NRS). The exclusion criteria included history of knee surgery or arthroscopy; pain in the knee caused by floating cartilage, joint effusion, inflammatory, malignant, or autoimmune disease; serious acute or chronic organic disease or mental disorder; pregnancy or breastfeeding; history of bleeding disorder. Participants were also not included if they had acupuncture treatment or participated in other clinical trials in the past three months.

Thirty participants matched healthy controls (HCs) were also recruited from October 10, 2018 to January 2019. The healthy subjects were between 45 and 75 years of age and had no pain or stiffness in both knees without major acute or chronic diseases. Eligibility data were entered on a secure online database and were monitored centrally before confirmation of study participation. The subject’s demographic data and medical history were obtained at baseline.

Prior to the study, the study process was explained to participants during recruitment. Participants were informed that participation in the trial was absolutely voluntary and that they can withdraw from the trial at any time. In the event of their withdrawal, collected data would not be deleted and were used in the final analyses. Otherwise, research investigators had to comply with Good Clinical Practice (GCP) guidelines in the study. No participant was included without full, written informed consent being first obtained. The study was approved by the medical ethical review committee of Beijing Hospital of Traditional Chinese Medicine Affiliated to Capital Medical University (2017BL-077-01) and was prospectively registered at ClinicalTrials.gov (NCT 03366363) on 20 November 2017, prior to recruitment of the first participant.

### Study Treatment

All acupuncturists in the study have Chinese medicine practitioner licenses; they have at least 5 years clinical experience. Hwato brand disposable, sterile steel needles (size 0.25×25-40 mm, manufactured by Suzhou Medical Appliance in Jiangsu, China) were used. Both EA and SA therapies consist of 24 sessions of 30 minutes, administered over eight weeks (usually three sessions per week).

#### EA Group

The obligatory acupoints included dubi (ST35), neixiyan (EX-LE5), ququan (LR8), xiyangguan (GB33) and an ashi point (the point where the patient felt most pain). Adjunct acupoints were chosen by the acupuncturists according to traditional Chinese medicine. If pain occurred in the anterior aspect of the affected knee joint, the patient had yangming meridian syndrome. Three adjunct acupoints were chosen from futu (ST32), liangqiu (ST34), heding (EX-LE2), zusanli (ST36) and fenglong (ST40). If pain occurred in the medial aspect of the affected knee joint, the patient had three-yin meridian syndrome. Three adjunct acupoints were chosen from xuehai (SP10), yingu (KI10), yinlingquan (SP9), xiguan (LR7), sanyinjiao (SP6), taixi (KI3), taichong (LR3) and gongsun (SP4). If pain occurred in the posterior aspect of the affected knee joint, the patient had taiyang meridian syndrome. Three adjunct acupoints were chosen from weiyang (BL39), weizhong (BL40), chengshan (BL57) and kunlun (BL60). If pain occurred on the lateral aspect of the affected knee joint, the patient had shaoyang meridian syndrome. Three adjunct acupoints were chosen from fengshi (GB31), yanglingquan (GB34), waiqiu (GB36), xuanzhong (GB39) and zulinqi (GB41). If more than two aspects were affected, three adjunct acupoints were chosen from those for the relevant syndromes. All acupoints were localized according to the WHO Standard. Needles were stimulated manually for 10 seconds to achieve “De Qi” sensation. An electrical apparatus (HANS-200A acupoint nerve stimulator, Nanjing Jisheng Medical Co, Ltd. production, density wave with frequency of 2/100Hz) would be then connected to the needles with alligator clips to stimulate the needles in pairs LR8-GB33 and 2 adjunct acupoints. The electric current was gradually increased until the needles began to vibrate slightly.

#### SA Group

Eight non-acupoints that were separate from conventional acupoints or meridians were used for the SA group. The schedule and other treatment settings were the same as for the EA group but with superficial skin penetration (2~3 mm in depth) and no electricity output or needle manipulation for de qi.

### Outcome Measures

#### Clinical Outcomes

The response rate was calculated according to a change of 50% from baseline in Western Ontario and McMaster Universities Osteoarthritis Index (WOMAC) total scores (pain, stiffness and function) at eight weeks (Witt et al., 2006). Knee pain were assessed by WOMAC pain subscale (five items, scored from 0 to 20) and NRS (scored from 0 to 10, 0 represent no pain, 10 represent unbearable pain) (Xie et al., 2008; Hawker et al., 2011). Stiffness and function were assessed by WOMAC stiffness (two items, scored from 0 to 8) and function subscale (17 items, scored from 0 to 68) (Xie et al., 2008). The standard 12-item Short Form Health Survey (SF-12, 0-100, higher scores representing better quality of life), an abbreviated form of the SF-36 that yields the physical and mental health summary (PCS and MCS), were used to assess the health-related quality of life of the participants (Ware et al., 1996).

### Sample Collection and DNA Extraction

Fecal samples were collected twice before and after treatment for KOA participants and once at baseline for HCs at hospital and then frozen at -80°C within three hours after sampling. Microbial DNA was extracted from human samples using the E.A.N.A.Ⓡsoil DNA Kit (Omega Bio-tek, Norcross, GA, U.S.) according to manufacturer’s protocols. The final DNA concentration and purification were determined by NanoDrop 2000 UV-vis spectrophotometer (Thermo Scientific, Wilmington, USA), and DNA quality was checked by 1% agarose gel electrophoresis. Demographics and clinical variables were collected during the clinic visits. Two cases were respectively excluded in EA group and SA group due to the change of defecation habits, such as diarrhea and constipation (EA group: GXY and WGD; SA group: LSL and CYH).

### 16S Ribosomal RNA Gene Sequencing

The V3-V4 hypervariable regions of the bacteria 16S rRNA gene were amplified with primers 338F (5’-ACTCCTACGGGAGGAGCAG-3’) and 806R (5’-GGACTACHVGGGTWTCTAAT-3’) by thermocycler PCR system (GeneAmp 9700, ABI, USA). The PCR reactions were conducted using the following program: 3 min of denaturation at 95°C, 27 cycles of 30 s at 95°C, 30s for annealing at 55°C, and 45s for elongation at 72°C, and a final extension at 72°C for 10 min. Amplicons were then purified by gel extraction (AxyPrep DNA Gel Extraction Kit, Axygen Biosciences, Union City, California, USA) and were quantified using QuantiFluor-ST (Promega, USA). The purified amplicons were pooled in equimolar concentrations, and paired-end sequencing was performed using an Illumina MiSeq instrument (Illumina, San Diego, California, USA).

### Microbial Analysis

The 16S rRNA sequencing data were processed using the Quantitative Insights Into Microbial Ecology platform (V.1.9.1). Sequencing reads were demultiplexed and filtered. Operational taxonomic units (OTUs) were picked at 97% similarity cut-off, and the identified taxonomy was then aligned using the Greengenes database (V.13.8). Chimeric sequences were identified and deleted. OTUs with a number of sequences<0.005% of the total number of sequences were removed from the OTU table. After filtering, an average of 34661 reads per sample was obtained (min: 23994; max: 42940). In addition, rarefaction was performed on the OTU table to prevent methodological artefacts arising from varying sequencing depth. Alpha-Diversity was measured by species richness from the rarefied OTU table. Beta-Diversity was estimated by computing bray-curits and was visualized with principal coordinate analysis. In efforts to dissect possible species for OTUs, we performed MegaBLAST search to align the reads of OTUs against reference sequences in the National Center for Biotechnology Information (NCBI) 16S rRNA database.

### Statistical Analysis

The clinical outcomes were analyzed using the SPSS software (SPSS V.12.0 KO for Windows). A value of *P*<0.05 would be considered statistically significant. Continuous data were expressed as mean and standard deviation (SD); enumeration data were expressed as a percentage.

The intestinal microorganisms were analyzed with R pack ages (V.2.15.3). For the comparison of continuous variables, Kruskal-Wallis test, Wilcoxon rank sum test and Wilcoxon signed-runk test were used. For correlation analysis, spearman’s rank test was performed. Multiple hypothesis tests were adjusted using Benjamin and Hochberg false discovery rate (FDR), and significant association was considered below an FDR threshold of 0.05.

## Results

Characteristics of treatment groups at baseline are presented in **Table 1**. There were no significant differences among three groups on any baseline demographic and clinical characteristics (all *P*>0.05).

**Table 1:** Demographic and baseline characteristics.

Characteristic	EA group (n = 30)	SA group (n = 30)	Healthy controls (n = 30)	P Value
Age (years), mean ± SD	64.73 ± 5.39	66.10 ± 7.42	63.67 ± 6.94	0.532
Women, n (%)	19 (63.3%)	20(66.7%)	19 (63.3%)	0.953
BMI (kg/m2), mean ± SD	26.04 ± 2.92	25.86 ± 4.02	24.58 ± 3.01	0.124
Education background (years), mean ± SD	12.07 ± 2.97	10.87 ± 2.87	11.73 ± 3.79	0.338
Bristol scores of stools, mean ± SD	4.17 ± 0.65	4.57 ± 0.57	4.50 ± 0.51	0.588

EA, Electroacupuncture; SA, Sham acupuncture; BMI, Body mass index was calculated as weight in kilograms divided by height in meters squared.

### Clinical Outcomes

After 8 weeks of treatment, the response rates (a change of 50% from baseline in WOMAC total scores) were 60% for EA group, higher than the 30% for SA group by intention-to-treat analysis (*P*=0.037) (**Table 2**).

**Table 2:** Response rate between EA and SA group at 8 weeks.

Outcome	EA group (n = 30)	SA group (n = 30)	P Value
Effective, n (%)	18 (60)	9 (30)	0.037
Non-effective, n (%)	12 (40)	21(70)	

EA, Electroacupuncture; SA, Sham acupuncture.

After eight weeks of treatment in **Table 3**, the total WOMAC score, WOMAC pain subscale, WOMAC stiffness subscale, WOMAC function subscale, and NRS score of the knee joint in the EA group (n=30) and the SA group (n=30) all decreased to varying degree. The quality of life score of SF-12 was improved both physically and mentally. At week 8, the NRS, WOMAC total score and WOMAC pain score in EA group were significantly lower than those in SA group (*P*=0.002, 0.043 and 0.026, respectively).

**Table 3:** Continuous outcomes at different week points.

Outcome	EA group (n = 30)	SA group (n = 30)	*P* value
WOMAC total scores			
Week 0	34.62 ± 10.39	33.96 ± 11.26	0.120
Week 4	24.97 ± 12.26	23.50 ± 12.58	0.649
Week 8	14.77 ± 7.11	20.23 ± 12.62	0.043
Week 16	19.33 ± 8.09	19.17 ± 11.07	0.947
Week 26	20.27 ± 8.06	19.33 ± 9.31	0.680
WOMAC pain			
Week 0	6.97 ± 2.68	7.00 ± 2.60	0.961
Week 4	4.87 ± 2.91	4.83 ± 2.98	0.965
Week 8	2.73 ± 1.72	4.17 ± 2.98	0.026
Week 16	3.73 ± 1.87	4.13 ± 2.71	0.509
Week 26	3.63 ± 1.67	4.03 ± 2.24	0.436
WOMAC stiffness			
Week 0	2.37 ± 1.27	2.47 ± 1.46	0.873
Week 4	2.03 ± 1.22	1.97 ± 1.33	0.840
Week 8	1.40 ± 1.00	1.87 ± 1.28	0.121
Week 16	1.50 ± 0.78	1.93 ± 1.11	0.085
Week 26	1.63 ± 0.96	1.73 ± 1.08	0.707
WOMAC function			
Week 0	21.63 ± 9.08	22.40 ± 8.25	0.468
Week 4	18.07 ± 8.94	16.70 ± 9.15	0.561
Week 8	10.63 ± 5.88	14.20 ± 9.65	0.089
Week 16	14.10 ± 5.96	13.00 ± 7.68	0.538
Week 26	15.00 ± 6.58	13.57 ± 6.52	0.400
NRS score (0–10)			
Week 0	6.00 ± 1.34	6.13 ± 1.33	0.700
Week 4	4.00 ± 1.82	4.10 ± 1.75	0.829
Week 8	2.00 ± 1.60	3.83 ± 2.53	0.002
Week 16	3.30 ± 1.20	3.70 ± 1.93	0.340
Week 26	3.13 ± 1.36	3.50 ± 1.70	0.359
SF-12 physical score			
Week 0	33.19 ± 6.84	33.58 ± 5.65	0.798
Week 4	34.63 ± 6.64	33.86 ± 6.69	0.647
Week 8	37.13 ± 5.57	35.22 ± 6.31	0.220
Week 16	37.05 ± 5.40	35.22 ± 6.85	0.241
Week 26	36.05 ± 4.31	35.33 ± 7.09	0.615
SF-12 mental score			
Week 0	41.62 ± 6.95	42.41 ± 8.24	0.704
Week 4	43.29 ± 6.22	43.04 ± 5.37	0.868
Week 8	43.86 ± 7.60	43.50 ± 6.74	0.856
Week 16	43.92 ± 5.96	42.90 ± 7.09	0.556
Week 26	44.09 ± 6.90	42.84 ± 6.41	0.484

EA, electroacupuncture; SA, Sham acupuncture; WOMAC, Western Ontario and McMaster Universities osteoarthritis index; SF-12, 12-item short-form health survey; NRS, Numerical rating scale.

Adverse events were uncommon and did not occur more frequently in either group. There was no significant bleeding in either group. During this trial, no participants took rescue medicine in both groups.

### Changes of gut Microbial Diversities in KOA

**Figure 1:**
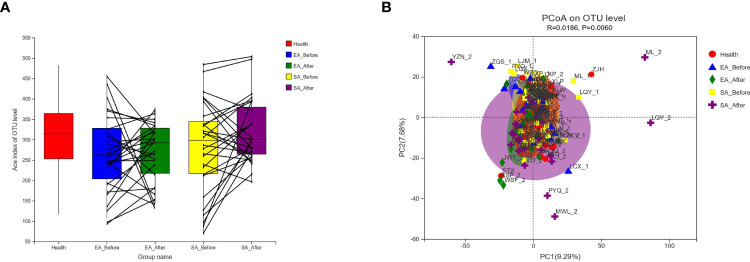
Changes of gut microbial diversities. **(A)** Alpha-Diversity in healthy controls, EA_Before group, EA_After group, SA_Before group, and SA_After group. Among the five groups, there was no statistical difference between each two group by Wilcoxon rank sum test. Alpha-Diversity, illustrated by microbiota richness [number of observed operational taxonomic unit (OUT)]. (Ace Index of Genus level in Wilcoxon rank sum test, *P* > 0.05) **(B)** An Bray-Curtis-based principal coordinated analysis (PCoA on Genus level in ANOSIM) between healthy controls, EA group and SA group with pre-post treatment (Beta-Diversity, *P* = 0.006).

As shown in **Figure 1A**, there was no significant difference among healthy controls, EA_Before group, EA_After group, SA_Before group, and SA_After group in the gut microbiota Ace index, measured by numbers of observed OTUs (*P*>0.05). A bray-curtis-based principal coordinated analysis revealed that the overall microbial composition in EA_Before group, EA_After group, SA_Before group, and SA_After group deviated with each other (*P*=0.006). The horizontal axis represents the first principal component (contribution rate is 17.47%) and the vertical axis represents the second principal component (contribution rate is 8.61%) (**Figure 1B**).

### Gut Microbial Dysbiosis in Treatment-Naïve KOA

**Figure 2:**
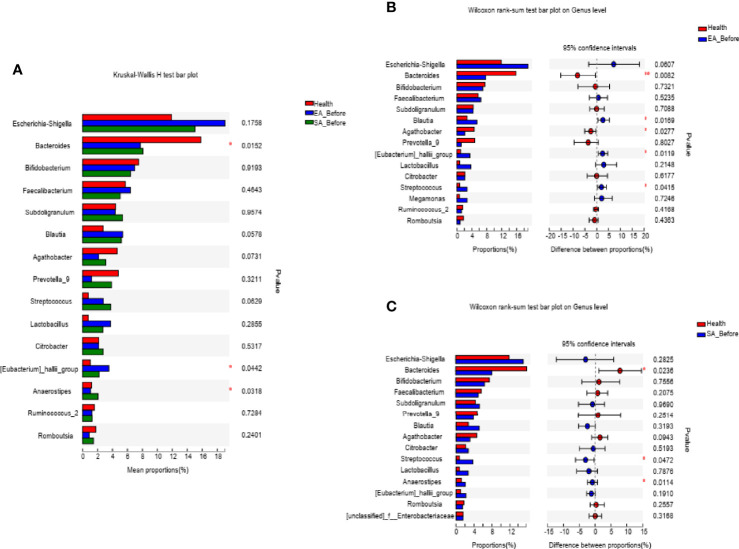
The barplots showed the relative abundance of genera enriched **(A)** among EA_before group (n = 28), SA_before group (n = 28) and healthy controls (n=30) (paired Kruskal-Wallis H test), **(B)** between EA_before group (n = 28) and healthy controls (n = 30) (paired Kruskal-Wallis H test), **(C)** SA_before group (n = 28) and healthy controls (n = 30) (Wilcoxon rank sum test). *P < 0.05, **P < 0.01.

At the genus level, 3 microbiota taxa (*Anaerostipes*, *Eubacteriumj_hallii_group*, *Bacteroides*) were found to have a changed relative abundance among EA_before participants, SA_before participants and HCs (Kruskal-Wallis H test) (**Figure 2A**). Furthermore, compared with HCs, 5 genera of EA_before group were significantly different, in which *Blautia* (*P*=0.0169*), Streptococcus* (*P*=0.0415), *Eubacteriumj_hallii_group* (*P*=0.0119) were increased, while *Bacteroides* (*P*=0.0082) and *Agathobacter* (*P*=0.0277) were decreased (Wilcoxon rank sum test) (**Figure 2B**). Compared with HCs, 3 genera of SA_before group were significantly different, in which *Streptococcus* (*P*=0.0472) and *Anaerostipes* (*P*=0.0114) were increased, while *Bacteroides* (*P*=0.0236) was decreased (Wilcoxon rank sum test) (**Figure 2C**).

### Electroacupuncture Treatment Partially Ameliorates Gut Dysbiosis of KOA

**Figure 3:**
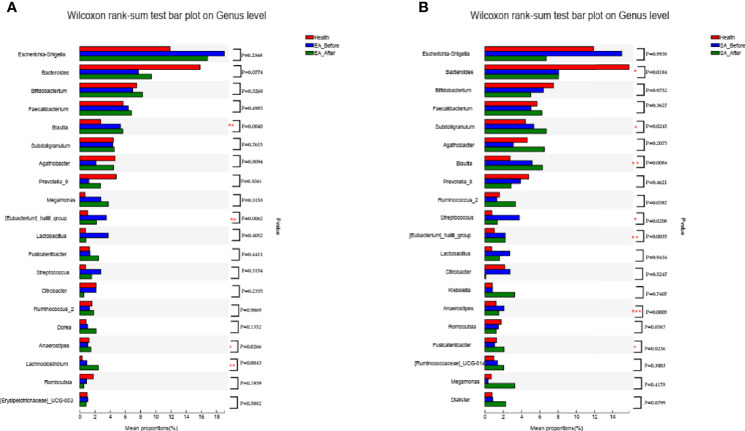
The barplots showed the relative abundance of genera enriched between **(A)** EA_After group (n = 28) and healthy controls (n = 30) (Wilcoxon rank sum test), **(B)** SA_After group (n = 28) and healthy controls (n = 30) (Wilcoxon rank sum test). *P < 0.05, **P < 0.01.

After EA treatment, four of the KOA-associated genera including *Bacteroides* and *[Eubacterium]_hallii_group*, *Agathobacte*r *and Streptococcus* were reversed, compared with HCs. The abundance of genera *Bacteroides* and *Streptococcus* were no different from that of HCs (**Figure 3A**). After SA treatment, two of the KOA-associated genera including *Agathobacter* and *Streptococcus* were reversed, compared with HCs, while the abundance was still different from that of healthy people (**Figure 3B**).

**Figure 4:**
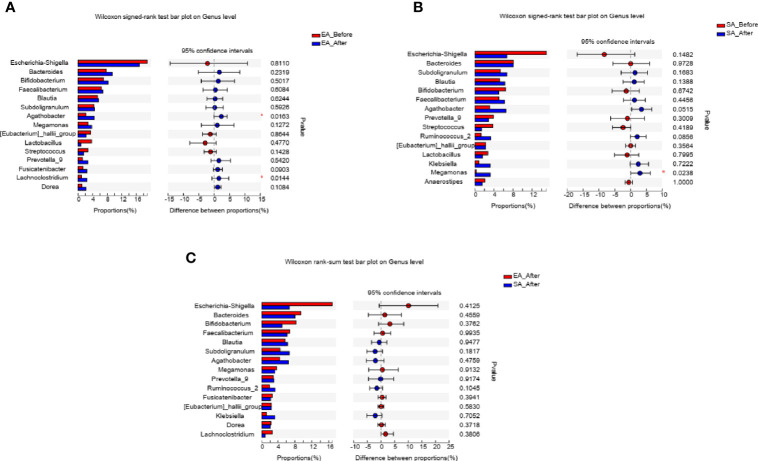
The barplots showed the relative abundance of genera enriched between **(A)** EA_Before (n = 28) and EA_After group (n = 28) (paired Wilcoxon rank sum test), **(B)** SA_Before (n = 28) and SA_After group (n = 28) (paired Wilcoxon rank sum test), and **(C)** EA_After group (n = 28) and SA_After group (n = 28) (Wilcoxon rank sum test). *P < 0.05.

After treatment, two genera including *Agathobacter* (*P*=0.0163), *Lachnoclostridium* (*P*=0.0144) were increased in EA_After group (paired Wilcoxon rank sum test) (**Figure 4A**). One genera (*Megamonas*, *P*=0.0238) in SA group had increased (paired Wilcoxon rank sum test) (**Figure 4B**). However, there was no difference in gut microbiome between EA_After group and SA_After group (**Figure 4C**) (Wilcoxon rank sum test).

**Figure 5:**
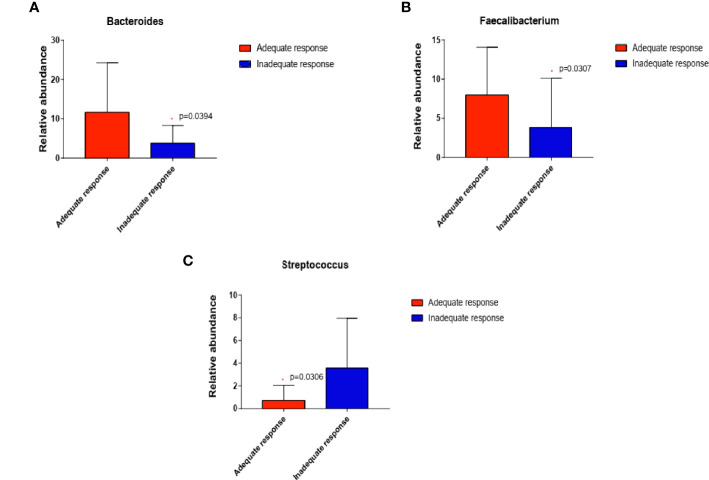
In EA_After group, the abundance of the three genera changed in patients who demonstrated adequate response than in those with inadequate response (n=20 and 8, respectively; **(A)** Bacteroides, P=0.0394; **(B)** Faecalibacterium, P=0.0307; **(C)** Streptococcus, P=0.0306) (Wilcoxon rank-sum test). *P < 0.05.

We then compared the relative abundance of KOA-associated genera between treated patients with an inadequate response to EA (WOMAC total score decreased by less than 50%) and those who demonstrated good improvement (WOMAC total score decreased by more than 50%). Two genera (*Bacteroides*, *P=*0.0394; *Faecalibacterium P=*0.0307) were more abundant while one genera (*Streptococcus*, *P=*0.0306) was significantly reduced in patients who demonstrated adequate response than in those with inadequate response (Wilcoxon rank-sum test) (**Figure 5**).

### Correlations Between the Gut Microbe and KOA Clinical Indices

**Figure 6:**
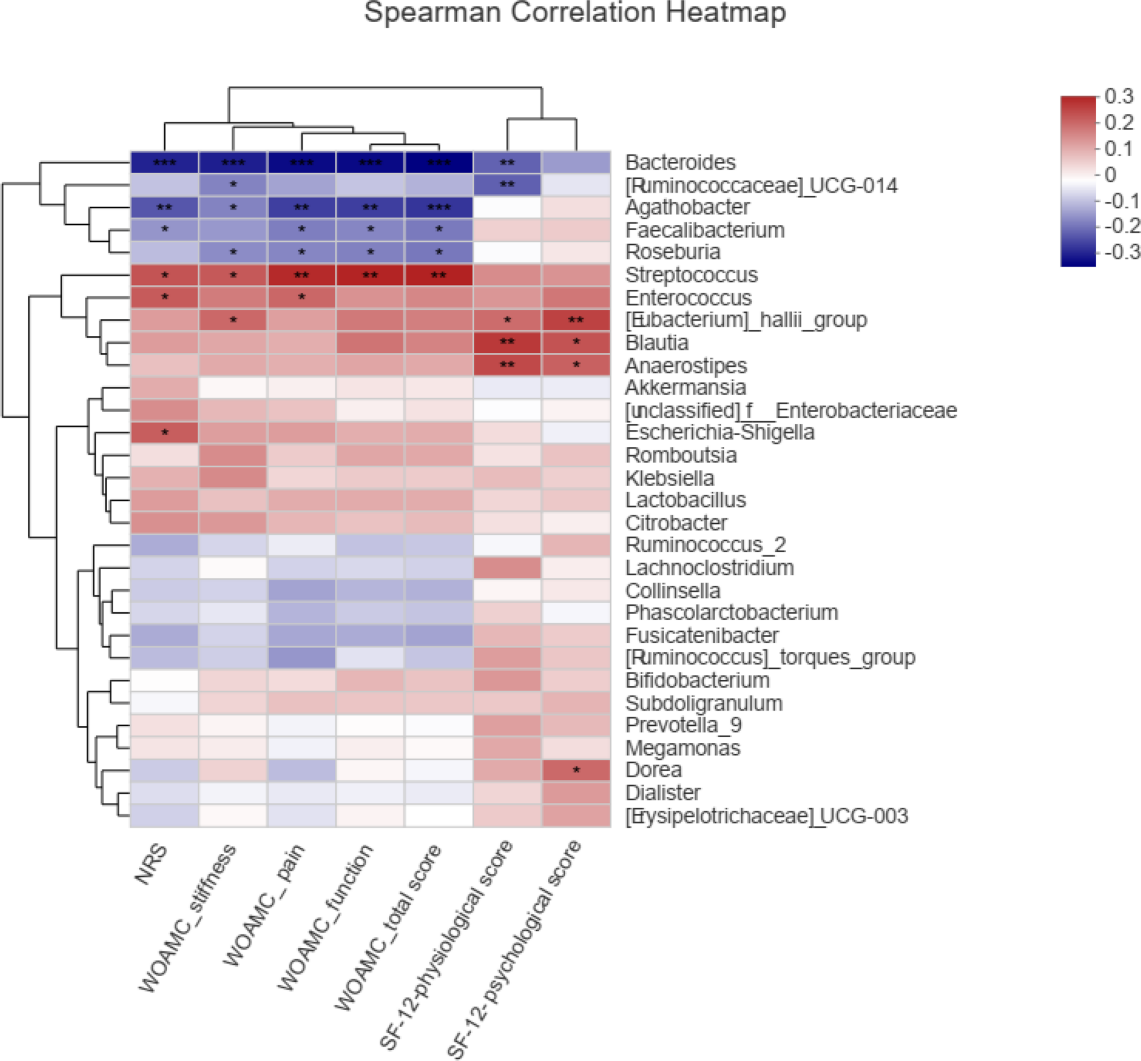
Spearman correlations between the genera and clinical indices including Western Ontario and McMaster Universities osteoarthritis index (WOMAC), Numerical rating scale/score (NRS), and Medical Outcomes Study 12-item short-form health survey (SF-12), *P < 0.05, **P < 0.01, ***P < 0.001.

Spearman correlation test was performed to evaluate the relationships between gut microbe and KOA clinical indices. **Figure 6** showed the top 30 genera, in which 6 genera were significantly related to NRS and WOMAC score. *Bacteroides* was negatively correlated with NRS score, WOMAC total score, and WOMAC pain, stiffness and function scores (*P*<0.001). *Agathobacter* was negatively correlated with NRS score, WOMAC total score, and WOMAC pain, stiffness and function scores (*P*<0.05 or 0.01). *Faecalibacterium* was negatively correlated with NRS score, WOMAC total score, and WOMAC pain and function scores (*P*<0.05). *Roseburia* was negatively correlated with WOMAC total score, and WOMAC pain, stiffness and function scores (*P*<0.05). *Streptococcus* was positively correlated with NRS score, WOMAC total score, and WOMAC pain, stiffness and function scores (*P*<0.05 or 0.01). *Enterococcus* was positively correlated with NRS score and WOMAC pain score (*P*<0.05). *[Eubacterium]_hallii_group*, *Blautia* and *Anaerostipes* were positively correlated with SF-12 physiological score and SF-12 psychological score (*P*<0.05 or 0.01).

## Discussion

In this study, we evaluated the effect of EA as a complementary therapy of knee osteoarthritis, with respect to relieve pain and microbiome compared with SA. The results suggested that EA was more effective than SA for pain associated with KOA in eight weeks. After treatment, the response rate of EA group was 60% and that of SA group was 30%, illustrating that the response rate of EA was 30% higher than that of SA.

Moreover, the treatment of EA modified the species richness and overall microbial diversity of gut microbiome, which is consistent with the results of the previous study (Xu et al., 2013). In our current study, we analyzed methods for sequencing 16S rRNA in KOA cohorts before and after acupuncture therapy. The results demonstrated that 8-week EA partially mitigated the microbial dysbiosis of KOA participants.

Then we applied a 16S rRNA sequencing approach to KOA cohorts before and after acupuncture therapy, compared with healthy controls. The results demonstrated that there was no significant change in the overall diversity of gut microbiota by alpha-diversity in KOA, compared with healthy controls. This might be related to the fact that the difference in microbial diversity between individuals was larger than that between groups, and a future study with adequate statistical power for finding differences in microbial diversity should be carried out. Base on beta-diversity analysis, EA could modify the composition of microbiome, which is consistent with the results of the present study (Xu et al., 2013). Combined with the comparison of microbial composition between KOA patients with pre-post treatment and healthy controls, we could find that EA could reverse more KOA-related bacteria, and after EA treatment, the different genera between KOA patients and healthy controls were less, compared with that after SA treatment. This meant that EA modified the composition of the gut microbiome, making it closer to healthy people.

The abundance of *Streptococcus* was increased in patients with KOA. We found that greater *Streptococcus* abundance leads to higher knee WOMAC pain through local joint inflammation. This suggests a role for the gastrointestinal microbiome in OA related knee pain and inflammation (Collins et al., 2015). It was found that *Streptococcus* abundance was significantly associated with effusion severity in the knee joints (Collins et al., 2015). This leads to a belief that *Streptococcus* might also be involved in other inflammatory joint pain disorders. The rate of inflammatory response in OA population is much greater than that in non-OA population (Puenpatom and Victor, 2009), and the KOA-enriched genera may thrive and contribute to inflammation or autoimmunity under inflammatory conditions. Then, in our study, *Streptococcus* was reversed in KOA after EA treatment, compared with SA treatment. We speculate that EA may rapidly reduce the signs and symptoms of osteoarthritis of the knee by altering the structure of the intestinal microbial community, particularly by reducing the *Streptococcus* abundance.

In addition, we found that the abundance of *Bacteroides* and *Agathobacter* were decreased in patients with KOA. *Bacteroides* and *Agathobacter* were negatively correlated with WOMAC total score, and WOMAC stiffness, function and pain scores. Many anaerobic intestinal microbiota such as species in *Bacteroides* and *Agathobacter* are known to produce short-chain fatty acids (SCFA) by fermentation of dietary fibers (Zhang et al., 2015; Koh et al., 2016; Hua et al., 2020). SCFA is known to exert a beneficial effect on health through the anti-inflammatory effects. Decreased production of SCFA by microbiota raises luminal oxygen concentration in mice, resulting in the expansion of facultative anaerobes (Kelly et al., 2015). SCFA-producing bacteria with elevated fecal SCFA concentrations may promote the energy intake from fibers, inhibit opportunistic pathogens and protect the hosts against inflammation and colonic diseases (De Filippo et al., 2010). EA is considered as a secure and effect tool for repelling pain (Zhang et al., 2014). In our study, when the pain was relieved after EA treatment, *Agathobacter* was significantly more abundant than before. *Bacteroides* was significantly more abundant in patients who demonstrated inadequate response than in those with adequate response. So we hypothesized that EA treatment could increase the abundance of beneficial bacterium *Bacteroide* and *Agathobacter*, and protect the hosts against inflammation, thereby achieving the purpose of relieving pain.

According to traditional Chinese medicine (TCM) theory, acupuncture produces therapeutic effects through acquiring *Deqi* manually. *Deqi* is a specific needle sensation, referring to the response to stimulations such as the thrusting, lifting, or rotating of the needle after insertion. It has been asserted to be a criterion to determine the appropriateness of acupuncture stimulation (Yin et al., 2015). According to syndrome differentiation, the lesions of 60 KOA patients in this study were mainly distributed on 4 meridians, among which the lesions with the syndrome of Foot Yangming Stomach Meridian were the most common (44 cases), accounting for 73.33%.

Although our investigations attempt to provide a comprehensive insight into the potential contribution of the gut microbiome in KOA, there are several limitations to be addressed in future studies. First, we were not able to observe variations among participants at different disease stages, probably due to a relatively small number of participants at moderate or advanced stage, as most participants in our cohort were Kellgren-Lawrence grade II or III (mild or moderate) radio graphically-confirmed KOA on one or both knees. Second, the small sample size in this trial increases the possibility of a type II error (i.e., a real effect of acupuncture being missed because of insufficient power). For future trials, sample size estimation could be calculated, for example, using PASS software, based on the data derived from this trial. Third, we did not validate the characteristics of the flora in an independent set of patients with KOA, and further investigation is needed to verify in a small independent cohort. Nonetheless, our comprehensive investigation of the gut microbiome in KOA reveals that compositional dysbiosis in participants were partially alleviated by EA. The identified KOA microbial signature needs further validation in larger cohorts.

## Conclusion

Our study suggested that EA could significantly relieve KOA pain. Although gut microbiome might not be related to KOA, EA could improve the inflammatory effects by changing serval genera in gut microbiota. In particular, EA reduced the abundance of pathogenic bacteria, such as Streptococcus and increased the abundance of beneficial bacteria including Agathobacter and Bacteroide in the gut. Although we have no direct evidence that the bacteria changed by EA can relieve KOA pain, our clinical research still provides indirect evidence that gut microbiota may be involved in the treatment.

## Data Availability Statement

The datasets presented in this study can be found in online repositories. The name of the repository and accession number can be found below: National Center for Biotechnology Information (NCBI) BioProject, https://www.ncbi.nlm.nih.gov/bioproject/, PRJNA659607.

## Ethics Statement

The studies involving human participants were reviewed and approved by Beijing Hospital of Traditional Chinese Medicine affiliated to Capital Medical University (2017BL-077-01). The patients/participants provided their written informed consent to participate in this study. Written informed consent was obtained from the individual(s) for the publication of any potentially identifiable images or data included in this article.

## Author Contributions

Study concept and design: C-ZL, T-QW, J-FT and L-RL. Acquisition, analysis, or interpretation of data: C-ZL, L-QW, J-WY, G-XS, Z-SL, HH, JW, TW, J-FT, C-XT and YY. Drafting of the manuscript: T-QW, J-WY, L-QW and W-RJ. Critical revision of the manuscript for important intellectual content: J-WY, J-FT, C-XT and L-QW. Obtaining of funding: C-ZL and J-FT. Study supervision: C-ZL and J-FT. All authors contributed to the article and approved the submitted version.

## Funding

Supported by grants from Beijing Municipal Administration of Hospitals Clinical Medicine Development of Special Funding Support (XMLX201607) and Beijing Municipal Science & Technology Commission (D171100003217003).

## Funding

This study was supported by Beijing Municipal Administration of Hospitals Clinical Medicine Development of Special Funding Support [XMLX201607] and Beijing Municipal Science & Technology Commission [D171100003217003]. The funders had no role in the study other than to provide funding. 

## Conflict of Interest

The authors declare that the research was conducted in the absence of any commercial or financial relationships that could be construed as a potential conflict of interest.

## Publisher’s Note

All claims expressed in this article are solely those of the authors and do not necessarily represent those of their affiliated organizations, or those of the publisher, the editors and the reviewers. Any product that may be evaluated in this article, or claim that may be made by its manufacturer, is not guaranteed or endorsed by the publisher.
